# A Method for the Preparation of Chicken Liver Pâté that Reliably Destroys Campylobacters

**DOI:** 10.3390/ijerph120504652

**Published:** 2015-04-28

**Authors:** Mike Hutchison, Dawn Harrison, Ian Richardson, Monika Tchórzewska

**Affiliations:** School of Veterinary Sciences, University of Bristol, Langford, Bristol BS40 5DU, UK; E-Mails: Dawn.Harrison@bristol.ac.uk (D.H.); Ian.Richardson@bristol.ac.uk (I.R.); Monica_Tchorzewska@yahoo.co.uk (M.T.)

**Keywords:** *Campylobacter*, pâté, effective cooking, food safety, critical control point

## Abstract

This study devised a protocol for the manufacture of commercial quantities of chicken liver pâté that reliably destroyed campylobacters. A literature search identified 40 pâté manufacture recipes. Recipes stages with a potential to be antimicrobial were assembled to form a new protocol that included washing with organic acid, freeze-thaw and flambé in alcohol. Naturally-contaminated, high-risk livers were obtained from clearance flocks at slaughter and the effect of each stage of the protocol on *Campylobacter* populations was determined. Organic acid washing changed the color of the liver surfaces. However, there were no significant differences between liver surface color changes when a range of concentrations of lactic acid and ethanoic acid washes were compared by reflective spectrophotometry. A 5% (w/v) acid wash reduced numbers of indigenous campylobacters by around 1.5 log_10_ CFU/g for both acids. The use of a Bain Marie was found to more reproducibly apply heat compared with pan-frying. Antimicrobial recipe stages reduced the numbers of campylobacters, but not significantly if thermal processing was ineffective. Cooking to 63°C was confirmed to be a critical control point for campylobacters cooked in a Bain Marie. Organoleptic and sensory assessment of pâté determined an overall preference for pâté made from frozen livers.

## 1. Introduction

Infection by *Campylobacter* spp. is responsible for the majority of cases of foodborne bacterial gastroenteritis in developed countries [1]. In England and Wales, the annual incidence of infection is typically around nine cases for each 10,000 of the population. Therefore, in 2012, there were more than 65,000 laboratory-confirmed cases, with an annual, seasonal peak of human infections in late spring [2]. However, only one in seven *Campylobacter* infections is reported [3], so the disease burden is likely to be significantly higher than the number of confirmed cases. In contrast to the trend of reducing numbers of infections by other foodborne bacterial pathogens such as *Salmonella* and verocytotoxic *E. coli*, within the European Union the incidence of campylobacteriosis has increased or remained unchanged each year over the last decade [4]. Investigations into the sources of campylobacters infecting humans have indicated a poultry reservoir [5], with molecular typing source attribution studies concluding that *Campylobacter* strains isolated from chicken livers were the most similar to human isolates [6].

A particular concern for the increasing incidence of campylobacteriosis is commercial caterers and restaurants, because if chicken livers are undercooked, large numbers of people can become infected [2,7,8]. In Scotland, an outbreak involving 48 people lasted three weeks and occurred after a single restaurant changed the method it used to cook chicken livers [7]. The likelihood that in the UK chicken livers will be contaminated with campylobacters is high. In northeast Scotland, a retail survey reported that 81% of chicken livers were contaminated [6]. More recently, more general surveillance across Great Britain reported that 87% of chicken livers purchased at the retail level were contaminated, with a geometric mean load of 2.2 log_10_ CFU campylobacters/g liver [9]. There are several reports that campylobacters can internally contaminate livers [10,11] and associated appendages such as gall bladders and bile ducts [12]. Internal contamination of liver with campylobacters means more than a surface sterilization is required to render them safe for human consumption [11].

Although it is evident that undercooked chicken livers can cause outbreaks of campylobacteriosis, it has been determined that >70 °C for at least two minutes at a liver’s core must be attained to cook livers safely [11]. An important consideration was that undercooked livers were pink, a coloration that had consumer appeal [11]. Furthermore, if livers were cooked for too long, they became unappetizing and grey with unfavorable changes in texture [11]. It has been suggested [8], and also unequivocally determined [7] that caterers had been undercooking livers in an attempt to keep them pink and appealing to consumers. At the same time, imperfect cooking had led to outbreaks of campylobacteriosis associated with the consumption of chicken liver dishes.

The purpose of this study is to decrease the food safety risks associated with the consumption of chicken livers. We report the findings of a thermographic assessment of the temperatures attained internally and externally during Bain Marie cooking and pan-frying of livers. In addition, we validate a Bain Marie-based cooking method that reproducibly destroyed campylobacters but kept the livers pink with an appealing texture. Finally, we undertook a step-by-step microbiological assessment and full sensory evaluation of a recipe for chicken liver pâté that was cooked using a Bain Marie to a validated campylobacteriocidal temperature.

## 2. Experimental Section

### 2.1. Identification of Chicken Liver Pâté Recipes and PROMPT Analyses

The chicken liver pâté recipe searches were undertaken by using the Google search engine using the search string “chicken liver pâté recipe”. In addition, recipes were sourced from cookbooks by visiting local libraries and bookstores. Initially, each recipe was reviewed and assessed for cooking method, the use of any antimicrobial ingredients and any antimicrobial process stages that might lower the number of campylobacter present in the chicken liver. Any recipe-specific factors that were likely to confound antimicrobial actions were also highlighted. Subsequently, two researchers independently scored the Provenance, Relevance, Objectivity, Method, Presentation and Timeliness (PROMPT) values of each recipe by assigning a score of between 0 and 5 for each attribute [13]. The mean score for both researchers and for each attribute were used as an index of the quality of the recipe, the author and any supplementary information. For inclusion, a mean PROMPT score of three or greater was required.

### 2.2. Liver Sources

For samples requiring microbiological assessment, fresh chicken livers were collected directly from slaughterhouses so that previously-thinned final clearance birds could be selected as those most likely to be colonized with high numbers of campylobacters. Furthermore, direct collection meant confidence that the livers had not been frozen previously and had been only routinely processed to remove gall bladders and bile ducts before cleaning and chilling in slush ice. Livers for human consumption were purchased fresh from a local food store.

### 2.3. Temperature Monitoring of Cooked Livers and Bain Marie Cooked Pâté

To measure the temperature of chicken livers during pan-frying, infrared thermography was undertaken using a radiometric thermal imaging camera (Fluke Ti25, Norwich, UK). The unit was calibrated by the manufacturer to have a measurement error of <2% of the temperature reading. An emissivity value of 0.85 was used for all images. The Smartview software program (version 3.1, Fluke) was used to process the raw camera data to thermographic images. Pan-frying was undertaken using butter (10 g) and a pan surface temperature of around 100 °C, to prevent excessive oxidation of the fat. For the determination of liver surface temperatures during pan-frying, thermographs of liver surfaces were taken after cooking for 100 sec on each side. Internal temperatures of the livers were assessed from thermal images of cut surfaces. Temperatures inside pâté cooked in a Bain Marie were directly monitored using temperature loggers set to record the temperature every minute (Tinytag Plus 2; Gemini Data Loggers, Chichester, UK). One logger was placed in each corner of a square terrine and another was placed in the center. A scaffold made from matchsticks was used to ensure there was no direct contact between the loggers and the base or sides of the terrine. The loggers were covered with pâté macerate. There was a 1.2 cm thickness over the top of the logger.

### 2.4. Liver Washing

Livers were washed by immersion in an equal mass of ethanoic or lactic acid, tap water or milk or selected combinations of these wash treatments. The concentrations of organic acid used ranged from 1%–5% (w/v; Table S1). The duration of the immersion for organic acids or tap water was two minutes at ambient (21 °C) temperature. Soaking in milk was undertaken for one hour at 4 °C with periodic agitation every 15 min.

### 2.5. Microbiological Examination

For each sample of raw liver or cooked pâté, a 25 g subsample was generated by chopping if required and then removing small randomly-selected pieces using a sterile teaspoon to ensure that liquid meat exudate was also sampled. An equal volume of maximum recovery diluent (MRD, Oxoid, Basingstoke, UK) was added to each liver sample before placing in a stomacher (Model number BA 6021, Seward, UK) and running for one minute to effect homogenization. *Campylobacter* spp. were enumerated using the ISO 10272 part 2 direct-plating method [14] with minor modifications. In brief, 2 mL of the initial 1:5 dilution were spread onto six plates of modified charcoal cefoperazone deoxycholate agar (CCDA, Oxoid CM0739 plus SR0155). The next dilution was a 1:10 and all subsequent dilutions were decimal as described by ISO 10272. All dilutions were made using MRD. 100 µL volumes of the decimal dilutions were plated in duplicate onto CCDA. Incubation was under microaerobic conditions (CampyGen, Oxoid) at 41.5 °C for 48 h.

Confirmation of *Campylobacter* spp. was by an inability of five colonies to grow in an aerobic atmosphere when streaked on Columbia Blood Agar (Oxoid) at 41.5 °C and a positive oxidase reaction for five colonies. In addition, a single colony from each plate was tested serologically for visible agglutination using Dryspot Campylobacter (Oxoid) according to the manufacturer’s instructions.

### 2.6. Reflective Spectrophotometry

Liver surface color was assessed before and after immersion in tap water, ethanoic or lactic acid for two minutes. Also assessed were immersion in 1% fat milk for one hour without and in combination with the acid and water immersions. Color measurement before and after treatment was undertaken using a Minolta Chromameter CR400 (Minolta Camera Company Ltd., Milton Keynes, UK), according to manufacturer’s instructions. Measurements of L* (luminance), a* (red-green), and b* (yellow-blue) values were undertaken in the CIELAB chromatograph as the average of three readings per liver assessed. There were at least two livers assessed per trial, before and after each immersion treatment and the trials were repeated on three occasions using different livers from different flocks.

### 2.7. Sensory Analyses

Panel assessors were selected and trained according to ISO 8586:2012 [14] and received additional training in the assessment of specific pâté characteristics as intensity scales. The descriptors used are listed in Table 1. In addition to sensory evaluation, hedonic descriptors of color likeability of sample, flavor likeability of sample and overall likeability of sample were scored also. A range of descriptors were selected from previous historic sensory evaluations and provided to the panelists. Thereafter, they discussed the words amongst themselves and decided which words to keep and the additional words they would like to use. All of the descriptors were assessed on a linear sliding scale from soft/smooth/nil to firm/coarse/extreme or in the case of color from pink to grey-brown. Tasters had a further two training sessions to familiarize themselves with the descriptors and intensities experienced. In each training session, tasters were given samples manufactured in the same way as the experimental material to be tested and a commercial preparation of pâté purchased from a local retailer.

On the day of the test panel, each panelist was given a sample from each of the two pâtés. The samples were randomly coded and supplied to each panelist in a different order. Four different batches of each treatment were assessed within a single morning. The random sample orders, replications and recording of assessment scores were managed using the Fizz software package (Biosystèmes, Couternon, Burgundy, France).

**Table 1:** Summary of the results of a sensory evaluation of pâté made from fresh and frozen livers undertaken by a professional taste panel. ^a^Standard Error of the Mean (SEM) of the individual taster scores

Attribute	Total Assessment Score for Pâté Made From		*p* Value	SEM ^a^
	Fresh Livers	Frozen Livers		
Color on surface	70.5	67.1	0.29	3.25
Color inside	28.7	47.6	<0.001	3.20
Color likeability	38.6	40.4	0.63	3.62
Texture on cutting	29.9	30.9	0.8	3.63
On-eating firmness	41.3	22.1	<0.001	4.43
On-eating dissolvability	73.9	75.1	0.72	3.31
Flavor strength	52.7	61.9	<0.001	2.21
Fatty	39.0	37.6	0.62	2.65
Livery	40.4	41.0	0.84	2.68
Acidic	8.4	12.8	<0.05	1.93
Rancid	3.6	4.2	0.45	0.69
Sweet	30.6	30.7	0.94	2.33
Peppery	21.3	22.3	0.7	2.63
Herby	29.9	33.1	0.2	2.55
Salty	22.2	22.8	0.71	1.49
Residue after eating	20.1	7.8	<0.001	2.84
Flavor likeability	44.8	51.0	<0.05	2.65
Overall likeability	36.0	48.6	<0.001	3.05

### 2.8. Statistical Analyses

Paired and homoscedastic t-tests, analysis of variance and Tukey’s honestly significant difference (HSD) *post hoc* analyses were undertaken using the SPSS software (Version 21; IBM, Armonk, NY, USA) and used to compare color data and the log10 numbers counted from the liver samples as appropriate. For all tests, a *p* value of <0.05 was used to determine any significant differences between treatments.

## 3. Results

### 3.1. Compilation of a Pâté Manufacture Protocol

A total of forty different chicken liver pâté recipes were sourced from books and numerous internet websites, including supermarkets’ own websites, celebrity chef websites or cookery blogs. An initial assessment revealed that most recipes suggested cooking livers using a frying pan and then typically, post-cook blending in a food processor. Importantly, the majority of those recipes stated a need to ensure that the pan-fried livers were still pink in the center. Alternative cooking methods identified included microwaving, poaching and pre-cook blending in a food processor before oven-baking in a Bain Marie. Potential antimicrobial ingredients included alcohol (35/40 recipes); onions or chives, which contain a number of heat-stable antimicrobial compounds (32/40); garlic, which contains an antimicrobial compound called allicin (30/40); pepper (26/40), which contains capsaicin and thyme, which contains the phenolic derivative antimicrobial compound, thymol (20/40). However, the majority of the recipes cooked the potentially antimicrobial ingredients prior to their contact with the livers, making it possible the activity of any antimicrobial compounds was reduced by thermal inactivation or vaporization [15]. Soaking livers in milk prior to cooking was included in three recipes, soaking in brandy in one recipe, while flambéing in brandy or other alcohol was included in three of the evaluated protocols. Each of those practices was perceived as a likely additional antimicrobial stage, which might result in a reduction to the number of campylobacters. From the twenty two recipes subjected to PROMPT analysis, only nine recipes had an average PROMPT score equal or higher than 3, and only three had an average score that was equal or higher than 3.5. The highest and lowest scored recipes are provided as Table S1.

### 3.2. Thermal Processing

**Figure 1:**
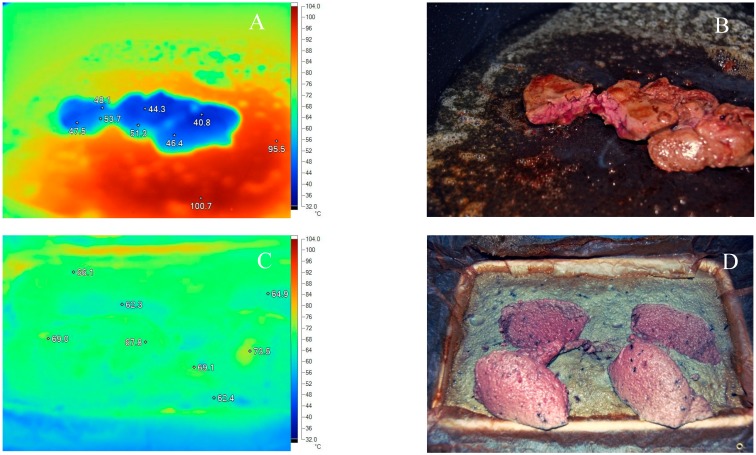
Thermographs showing surface and internal temperatures (**A** and **C**) and corresponding visible images showing color and appearance (**B** and **D**) after cooking by pan frying in butter on a surface at 100 °C for 100 sec on each side of the liver (**A**) or in a Bain Marie of boiling water placed in an oven set to 130 °C for 45 min (**B**). Both sets of cooked livers were subject to 10–15 sec of cooling before the thermographs could be taken. The Bain Marie cooked pâté set only after cooling and was subject to rheological movements between image captures.

The cooking method for the pâté manufacture protocol was chosen after undertaking thermographic assessment of cooked livers (Figure 1). In keeping with the previous observations of other authors [11], pan-frying resulted in an uneven heat application. The centers of the pan-fried livers were commonly significantly cooler than the surfaces. Once removed from the pan surface, the livers cooled rapidly as has been previously reported [11]. We measured a cooling rate of 0.5 °C/sec on average between 70 °C and 45 °C. In contrast, cooking using a Bain Marie required around 45 minutes for the core temperature to reach 68 °C.

**Figure 2:**
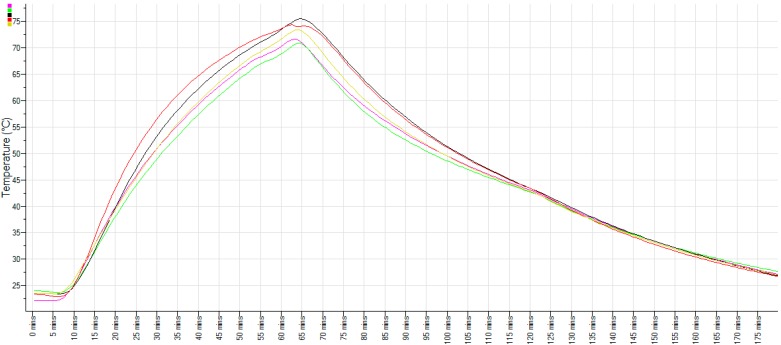
Typical temperatures achieved at the corners (yellow, black, red and green) and center (pink) of a terrine of macerated chicken livers during baking in a Bain Marie and during cooling after removal from the oven.

In the Bain Marie, the longer heating time allowed the core temperature to rise slowly and resulted in an apparent more uniform temperature throughout the mass of the pâté (Figure 1C). However, once removed from the oven, the exposed surface of the Bain Marie-cooked pâté also cooled rapidly. The image shown in Figure 1C was taken around 10 sec after removal from an oven set to 130 °C. In order to accurately measure the temperature inside the pâté, temperature loggers were placed at the corners and center of a square terrine and baked with the liver macerate. A typical set of temperature traces is shown as Figure 2. There were no significant differences when the temperature records from each logger were compared during the time interval that cooking occurred, or the time interval that the pâté was cooking and cooling (both ANOVA, *p* > 0.05). As might be expected, the most rapid heating was observed to be in the two corners of the terrine closest to the oven fan. The center of the macerate and the corner of the terrine furthest from the fan had the lowest rate of heating and lowest final temperatures.

### 3.3. Effect of Washing

Washing with either of the organic acids assessed, caused changes to the color of the liver surfaces (Table S2). Typically, the liver surfaces were bleached by the acids and there was a reduction in the red component of the color reading (Table S2). Overall, there was no apparent correlation between acid concentration and the degree of bleaching, which may have been a consequence of livers from different flocks having slightly different colors, and being bleached with varying degrees. Irrespective if that was the case, cutting into acid-treated livers showed that the bleaching was confined only to the liver surface and extended less than 1mm into the livers. There was no significant difference between the colors of acid-treated and control livers after blending into a macerate.

**Figure 3:**
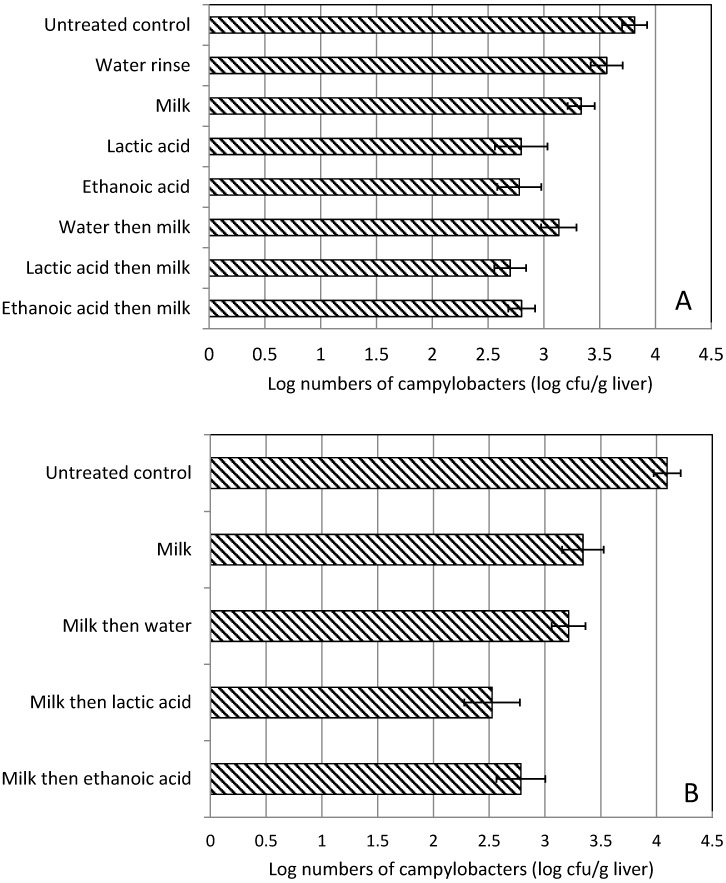
(**A**) The effect of immersing livers in water or organic acid (2 min for each solvent), milk (for 1 h) or organic acid immersion (2 min) followed by milk immersion (1 h) on the numbers of campylobacters present on chicken livers. (**B**) The effect of immersing livers in milk (for 1 h) followed by immersion in water or organic acid (2 min for each solvent) on the numbers of campylobacters present on chicken livers. For both experiments, final clearance livers were stored chilled at 2 °C for 48 h before the commencement of each treatment. Error bars are the standard error of the mean log count for both graphs.

Our initial strategy was to reduce the numbers of campylobacters contaminating the livers as quickly as possible, to reduce the likelihood of contamination of the kitchen environment. Therefore, for the initial experiments, chicken livers were subjected to organic acid washes before soaking in milk (Figure 1). However, the organic acid residues led to curdling of the milk and interfered with blood removal and therefore the experiment was repeated using the milk soak stage before the acid soak, with a tap water rinse in-between. The results showed that a wash with a 5% (v/v) solution of lactic or ethanoic acid significantly reduced number of campylobacters on the chicken liver surface, compared with untreated control or a water rinse (Table S3). Similarly, when the acid solution wash was followed by a milk soak, the reduction in *Campylobacter* numbers was significantly greater when compared with untreated control or water rinse for both lactate and ethanoate (Table S3). Typically, the reduction was around one log CFU/g (Figure 3A).

When the order of the combination treatment was switched from organic acid or water followed by a milk soak, to a milk soak followed by organic acid or water, the observations were that all of the treatments including milk soak-only significantly reduced *Campylobacter* numbers when compared with an untreated control (*p* < 0.001). For a milk soak followed by a lactic or ethanoic acid treatment, the livers contained significantly lower numbers of campylobacters compared with milk-only soaked livers (Figure 3B). There was no significant difference between the organic acid washes applied after the milk soak treatments.

### 3.4. Preparation of Chicken Liver Pâté

Pâté was prepared from fresh or freeze-thawed livers so that microbiological and sensory comparisons could be made between the two source materials. After the identification of likely antimicrobial stages and thermographic assessment of the different thermal processing methods, a protocol for the manufacture of chicken liver pâté was assembled and an illustrated version is provided as supplementary material.

In overview, to remove blood from the livers, 500 g were soaked in 500 mL of 1% fat milk containing a crushed garlic clove, two sprigs of bruised thyme, two bay leaves and 1% (w/v) salt. After the soak, the livers were rinsed in tap water and immersed for two minutes in 500 mL of store-bought vinegar, which was a 5% (v/v) solution of ethanoic acid. After draining, 50 mL of brandy was poured onto the livers, which were stirred to ensure an even coating of alcohol and the alcohol was ignited using a blow torch. The livers were mixed with a chopped clove of garlic and 120 g of chopped shallots that had been cooked by frying in a pan containing 50 g of melted butter with a surface temperature of 100 °C for 6 min. Beetroot (10 g) was added to the mixture to confer a red cast. The ingredients were liquidized in a blender (Model R2, Robot Coupe Ltd, Isleworth, Middlesex, UK). Whilst blending, five raw eggs and 450 g of melted butter were added to the mixture. The mixture was passed through a sieve (0.212 mm mesh; Cole Palmer, London, UK) to remove fat and connective tissue, and cooked in a Bain Marie of boiling water placed in an oven preheated to 130 °C. Although 70 °C has been previously reported as a safe cooking temperature for pan fried livers, the Bain Marie took much longer to heat the livers and consequently the pâté was baked until the temperature in the core reached 68°C, determined by thermocouple (Model C9007; Comark Instruments Ltd., Norwich, UK).

**Figure 4:**
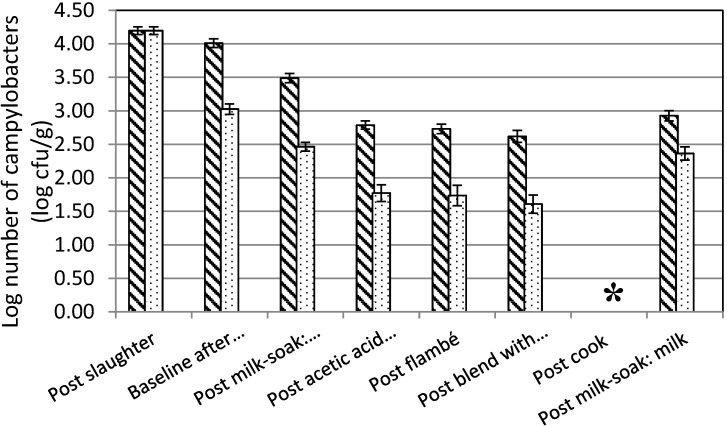
The numbers of campylobacters present in chicken livers at each stage of a protocol used for the manufacture of chicken liver pâté. Livers were either stored chilled at 2 °C for 48 h (diagonal hatch) or frozen to −20 °C for 24 h and thawed for 24 h (speckled dots) before the commencement of pâté manufacture. Error bars are the standard error of the mean log. An asterisk (*) denotes a sample that did not contain campylobacters determined by enrichment.

As was previously reported, campylobacters were present on livers from colonized final clearance flocks at roughly 10^4^ CFU/g [9,11]. In general, each stage of the manufacturing protocol reduced the numbers of campylobacters either by an antimicrobial action or by dilution caused by the addition of additional ingredients (Figure 4). Freezing and rinsing in vinegar caused significant reductions in the numbers of campylobacters (Tukey HSD, *p* < 0.05). Non-significant reductions of around one log were observed as a result of the milk soak, with larger-than-expected numbers of up to three log CFU/mL of campylobacters released into the milk. We speculate that small numbers of individual cells released from biofilm colonies could be the source of the elevated counts. Flambé in alcohol or blending with an equal mass of butter were relatively ineffective at reducing the numbers of campylobacters per g of liver or pâté (Figure 4).

**Figure 5:**
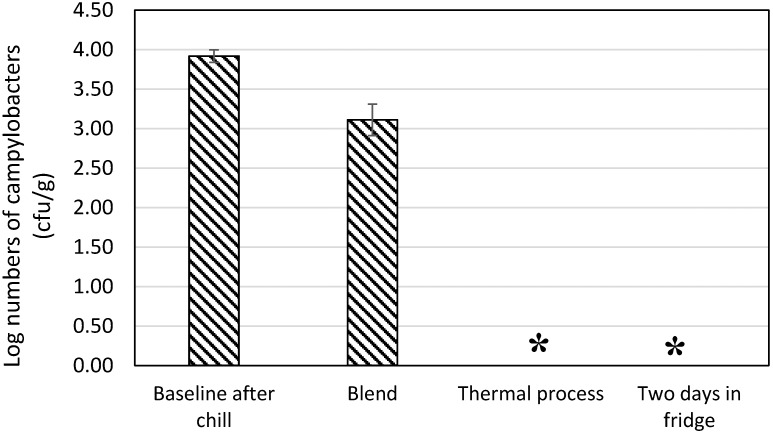
The numbers of campylobacters present in chicken livers before and after cooking in a Bain Marie under worst-risk conditions. Fresh livers from final clearance birds were prepared only by blending with an equal mass of molten butter before cooking and refrigerated storage to determine recovery from sub-lethal injury. An asterisk (*) denotes a sample that did not contain campylobacters determined by enrichment.

In order to unequivocally validate cooking in a Bain Marie of boiling water placed in an oven at 130 °C for ~45 min as a true critical control point, pâté was manufactured under worst case conditions of using fresh livers taken from final clearance flocks without any refrigerated storage, omitting any milk or acid soaks and without exposure to alcohol, flambé or the inclusion of any herbs. Livers blended with egg and butter and cooked in a Bain Marie as described above did not contain countable numbers of campylobacters (Figure 5).

Furthermore, when the pâté was enrichment tested four hours after cooking and after a further 48 h of refrigerated storage, no campylobacters were isolated (Figure 5). Thus, there was no evidence that the Bain Marie cooking caused only sub lethal injuries to the campylobacters and that recovery was possible.

**Figure 6:**
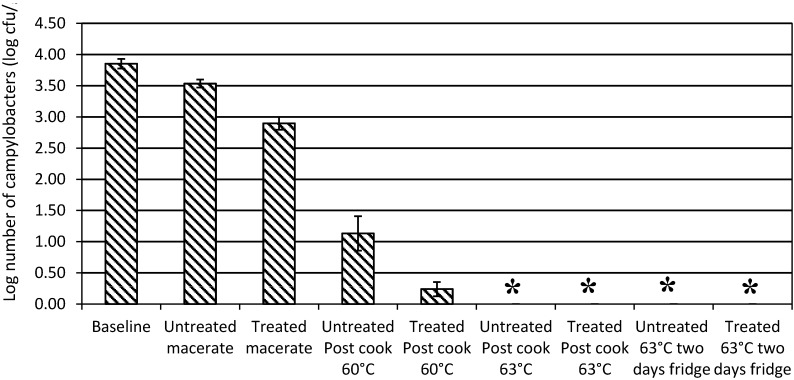
The numbers of campylobacters remaining after imperfect cooking of contaminated livers to 60 °C or 63 °C. Livers were treated by washing in organic acid and exposed to essential oils (EO) and compared with livers that were not washed in organic acid or exposed to EO to determine if treatment conferred additional safeguards. Error bars are the standard error of the mean log. An asterisk (*) denotes a sample that did not contain campylobacters determined by enrichment.

Experiments to determine an effect for an exposure to organic acid and essential oils prior to imperfect cooking, showed that there was a significant reduction to the treated macerate compared with the baseline livers (Figure 6). There was no corresponding significant reduction for the untreated macerate. After a 60 °C cook, although we observed that the treated macerate had lower numbers of campylobacters compared with the untreated counts, the improved reduction was not significant (ANOVA, Tukey’s HSD). No campylobacters were enumerated or detected by enrichment when cooking was to 63 °C for either the treated or untreated macerate. There was no evidence of recovery from sub-lethal injury after cooking to 63 °C followed by two days of refrigerated storage (Figure 6).

The results of the sensory comparison of pâté manufactured from fresh and frozen livers are summarized in Table 1. There were significant differences (*p* < 0.05) for the internal color of the pâté, firmness on eating, strength of the flavor, the amount of residue after eating and acidity for the two treatments. Both pâtés were assessed as acceptable by the taste panel, with a preference for pâté prepared from frozen livers.

## 4. Discussion

Previously, other studies have reported difficulties in assessing surface temperatures of chicken meat that was fried or boiled [16]. Our strategy to circumvent the issue was to use a radiometric device to determine the temperature profiles. Previously, it was determined that chicken livers reached around 70 °C at the core after two or three minutes of cooking [17]. However an important previous observation was also that the temperature in the cores and surfaces of livers dropped rapidly when they were no longer in contact with the pan surface [11]. Our contribution to these ongoing studies regarding the effective cooking of liver is that pan frying for 100 sec each side in a pan with a surface temperature of ~100 °C did not always uniformly heat the liver cores to 70 °C. Although the thickness of the livers will certainly influence heating and cooling rates, liver pieces of roughly 40 g mass still contained blood after cooking (Figure 1B). For that reason, the pate manufacture protocol used a Bain Marie for cooking, since it heated the mass of livers more reliably (Figure 2). If a probe was used to verify the temperature after cooking, it would be safest to insert it into the center of the pâté, where the temperature achieved was lowest. We consider that there was likely to be residual kill prior to refrigerated storage during the few hours it took for the pâté to cool to room temperature (Figure 2). Potentially the residual heat was the reason that 63 °C was sufficient to kill all of the campylobacters when previous workers had reported 70 °C or greater was required [11].

A number of different combinations of washing for the livers that involved tap water, milk and organic acid were undertaken. The greatest reductions to the numbers of *Campylobacters* on livers were for the treatments that included the organic acids. Although treatment with milk followed by lactic acid, showed the greatest reduction, ethanoic acid (vinegar) was selected for inclusion into the recipe because the cost of vinegar was significantly less than food-grade lactic acid. Furthermore, in contrast to lactic acid, vinegar is widely available from food stores. An observation made during treatments undertaken for Figure 3A dictated a change to the originally-compiled recipe. If an organic acid wash was applied prior to any milk soak, there was curdling of casein observed on the liver surfaces after the milk was removed. In addition, washing in acid interfered with blood removal from the livers during the milk soak. Post-soak milk was noticeably pinker for livers that had not been previously washed in acid. We speculate that the acid denatured surface liver proteins and thereby interfered with the osmotic removal of hemoglobin or red blood cells from the livers. In addition, post soak milk was pinker for frozen livers compared with fresh ones, which was most likely a consequence of the more straightforward osmotic removal of hemoglobin from freeze-ruptured erythrocytes.

Figure 4 shows the effect of each of the different recipe stages on fresh and frozen-thawed livers contaminated with *Campylobacter*. In general, each stage of the manufacturing protocol reduced contamination of the livers. The benefits of an initial freeze thaw of the livers in terms of reducing contamination by campylobacters are marked, confirming our previous studies [9]. In contrast, our observation that initial exposure to brandy followed by ignition of the alcohol was relatively ineffective at reducing campylobacters was surprising. Although previous studies have reported that exposure to 70% ethanol for 1 min did not reliably surface sterilize livers [18], there was a significant amount of heat generated whilst the alcohol burned. One potential explanation for the result was the ignition was typically only for a few seconds, which may have been insufficient to impact significantly on the *Campylobacter* populations. General studies assessing responses to heat have shown that refrigerated storage of chicken meat prior to heat exposure increases resistance to thermal processing [16]. Also of potential relevance is motility, which can be an important factor for *Campylobacter* survival by virtue of movement into favorable environments. *Campylobacter* cells were reported by [19] to move up to 3 mm inside crevices in chicken skin to avoid direct exposure to aerobic atmospheres. There are reports of *Campylobacter* isolations from the internal tissues of chicken livers and those campylobacters inside the livers [18] may have been protected from the short application of heat.

The addition of butter and eggs during blending essentially doubled the mass of the uncooked pâté. Consequently, we would have expected the numbers of campylobacters per g of material to reduce by half. The real number reductions were of the order of 20%. One possible explanation for this finding was that the campylobacters were present in the form of biofilms or colonies on the liver surface and the shear associated with blending disrupted these structures leaving at least some of the cells viable. There is evidence that campylobacters can bind and interact with specific eukaryotic cell surface receptors [20], which may provide the framework for close-knit growth. Traditional microbiological testing using stomaching has been assessed as effective in removing cells from biofilms compared with other methods in aqueous environments [21]. However that does not mean that stomaching removes all of the cells from a biofilm or that it provides an accurate measurement of bacterial numbers from such sources.

It should be kept in mind that the herbs that were included in recipe are not analytical grade reagents and the concentrations of essential oil antimicrobials will vary between different batches of plants [22]. Potentially, the issue could result in a variable decontamination performance for the marinade. Garlic contains the short-lived antimicrobial allicin [23] and there are heat stable antimicrobial compounds in shallots [24]. Thymol, an essential oil present in thyme, has strong antimicrobial activity, although it is poorly soluble in water [25]. We observed reductions in the numbers of campylobacters after the milk and herb soak of around one log for both fresh and frozen livers. However, at least part of the reduction was likely to be a consequence of the release of reversibly bound campylobacters from the liver surface into the milk [26], rather than true death. Evidence to support that hypothesis, and that the essential oils in the herbs were not able to cause reliable reductions to the numbers of campylobacters was provided by the observation that the spent herb-infused milk used for the soak contained viable campylobacters (Figure 4).

There were no significant differences between the numbers of campylobacters in livers treated with organic acid and essential oils and untreated livers when cooking was to 63 °C or 60 °C (Figure 6). However, these studies provided evidence that the use of a Bain Marie allowed the pâté to cool slowly over the course of a few hours prior to refrigerated storage and there was likely to be residual death of campylobacters during these initial stages of cooling.

As a likely consequence of the increased removal of hemoglobin from pâté made from frozen livers during the milk soak, fresh livers pâté was assessed by sensory evaluation as pinker than pâté from frozen livers. However, the color difference did not significantly influence color likeability. Frozen livers pâté was also less firm on eating but assessed as having a stronger flavor. For the descriptors used, the only significant difference between individual flavor traits for the two livers was acidity, with the frozen livers’ pâté being more acidic, although there was less residue after eating the pâté made from frozen livers (Table 1). Despite the increased acidity, pâté made from frozen livers was preferred for flavor and liked best overall by the nine panelists. A number of the recipes that used a milk soak stated that blood was removed to prevent bitter flavor in pâté and thus the improved blood removal in the freeze-thawed livers may have contributed to the favorable assessment. In addition to the more favorable sensory evaluation for frozen livers, there are additional benefits for freezing in terms of food safety. We have previously reported that freeze-thawing livers reduced the numbers of campylobacters by up to two logs [9]. Furthermore, it has been reported previously that refrigerated storage of contaminated chicken meat before cooking increased the heat resistance of a number of food borne human pathogens including *C. jejuni* [16].

## 5. Conclusions

Although there have been a number of outbreaks of campylobacteriosis that have been traced back to inadequately cooked chicken liver pâté [2,7,8], this study has determined it is straightforward to prepare commercial quantities of pâté that are not a risk to public health. Adequate cooking to 68 °C of highest risk livers sourced from final clearance flocks, is sufficient to destroy all campylobacters, even those internalized in livers. Cooking a Bain Marie resulted in pâté that was pink in color with other organoleptic properties considered desirable by caterers.

## Supplementary Files

Supplementary File 1
