# Supplementary material for: A Method for the Preparation of Chicken Liver Pâté that Reliably Destroys Campylobacters
Source: Int J Environ Res Public Health. 2015 Apr 28;12(5):4652–69. doi: 10.3390/ijerph120504652 (PMC4454932; doi:10.3390/ijerph120504652)
Supplement: Supplementary File 1 [file ijerph-12-04652-s001.pdf]

A Method for the Preparation of Chicken Liver Pâté that Reliably Destroys Campylobacters

An example summary of the PROMPT appraisal using the highest and lowest scored recipes

Table S1. A Provenance, Relevance, Objectivity, Method, Presentation and Timeliness (PROMPT) analysis of highest and lowest scored recipes.

Recipe Source	Attribute	PROMPT Analyses Notes	PROMPT Score
[1]	P	Author not identified.	1
	R	Most antimicrobials in whisky/brandy and peppercorns are cooked and therefore denatured/have reduced activity before any contact with the livers.	1
	O	No obvious bias for the All About You website. No branded ingredients were used. No branded products for complementary foods such as the recommended accompanying wine.	5
	M	Livers cooked until there is no blood left (5–10 min), clearly stated to not overcook them, but no reference to leave them pink in the middle.	2
	P	A list of all required ingredients was provided. Information regarding nutritional values included. Clearly laid out recipe-each stage was numbered. No pictures, photos or video, but the wording was descriptive enough to be easy to follow.	4
	T	Present on the website in 2012, relatively new recipe, however it does not contain anything that is new or novel.	1
		Average PROMPT score	2.33
[2]	P	Clearly-identified author with appropriate background/training/experience, a celebrity chef.	5
	R	Most antimicrobials in the garlic, shallots, thyme, madeira, port and pepper are cooked and therefore denatured/have reduced activity before any contact with the livers.	1
	O	No obvious bias for either the BBC website (which has no advertising) or Raymond Blanc. No branded ingredients were used. No branded products for complementary foods such as the recommended accompanying wine.	5
	M	Milk marinade will remove roughly half of the reversibly-bound campylobacters<br>Low cooking temperature of 70 °C.<br>But prolonged cooking duration of up to one hour.<br>Possible to set an objectively-measured target temperature setting for the oven.	4
	P	A list of all required ingredients was provided. Clearly laid out recipe-each stage was numbered. No pictures, photos or video, but the wording was descriptive enough to be easy to follow.	4
	T	No date provided for the recipe. Fairly novel use of a Bain Marie for cooking could be used to promote food safety compared with pan frying.	3
		Average PROMPT score	3.67

Table S2. Color changes to the surfaces of fresh chicken livers as a consequence of immersion ethanoic or lactic acids of different concentrations. [a] denotes the Standard Error of the Mean of six average color attribute scores. L, a, b, C and H are coordinates on a chromaticity diagram provided by the instrument manufacturer. In combination, they describe the measured color.

Treatment	Mean Color Component Change after Treatment (± SEM [a])					Overall Color Change
	ΔL *	Δa *	Δb *	ΔC *	ΔH *	(ΔE $*_{ab}$)
Water rinse	1.13 ± 0.22	−1.11 ± 0.33	−1.19 ± 0.25	−1.33 ± 0.38	0.93 ± 0.19	2.16 ± 0.27
1% ethanoic acid	5.24 ± 0.57	−2.64 ± 0.41	−1.11 ± 0.50	−2.88 ± 0.52	0.68 ± 0.26	6.15 ± 0.54
2% ethanoic acid	5.62 ± 0.40	−2.43 ± 0.37	−0.20 ± 0.20	−2.39 ± 0.35	0.61 ± 0.17	6.17 ± 0.49
3% ethanoic acid	4.64 ± 0.98	−2.61 ± 0.39	−0.45 ± 0.20	−2.65 ± 0.42	0.33 ± 0.08	5.37 ± 0.53
4% ethanoic acid	4.51 ± 0.45	−1.98 ± 0.17	−0.02 ± 0.31	−1.91 ± 0.15	0.74 ± 0.22	5.00 ± 0.43
5% ethanoic acid	3.19 ± 0.51	−1.11 ± 0.27	0.11 ± 0.20	−1.05 ± 0.31	0.39 ± 0.16	3.44 ± 0.53
1% lactic acid	2.90 ± 0.46	−2.70 ± 0.22	−1.85 ± 0.21	−3.04 ± 0.25	1.22 ± 0.18	4.47 ± 0.37
2% lactic acid	3.26 ± 0.55	−1.64 ± 0.48	−1.34 ± 0.36	−1.94 ± 0.56	0.83 ± 0.24	4.16 ± 0.47
3% lactic acid	2.12 ± 0.30	−1.60 ± 0.38	−0.73 ± 0.41	−1.74 ± 0.47	0.73 ± 0.15	3.00 ± 0.35
4% lactic acid	1.97 ± 0.45	−1.78 ± 0.31	−0.72 ± 0.41	−1.93 ± 0.39	0.73 ± 0.20	3.10 ± 0.26
5% lactic acid	1.59 ± 0.54	−0.96 ± 0.11	0.10 ± 0.08	−0.91 ± 0.12	0.34 ± 0.07	2.08 ± 0.38

Table S3. Tukey HSD (honest significant difference) comparisons for mean log campylobacter numbers after wash treatments.

Treatments Compared	*p*-Value	Accepted?
Acetate then Milk *vs*. Acetate	0.091069802	rejected
Acetate then Milk *vs*. Control	7.82 E−05	accepted
Acetate then Milk *vs*. Lactate then Milk	0.999601351	rejected
Acetate then Milk *vs*. Lactate	0.004568831	accepted
Acetate then Milk *vs*. Milk	7.82 E−05	accepted
Acetate then Milk *vs*. Water	7.82 E−05	accepted
Acetate then Milk *vs*. Water then Milk	0.9803316	rejected
Acetate *vs*. Control	7.82 E−05	accepted
Acetate *vs*. Lactate then Milk	0.243388561	rejected
Acetate *vs*. Lactate	0.921310888	rejected
Acetate *vs*. Milk	7.82 E−05	accepted
Acetate *vs*. Water	7.82 E−05	accepted
Acetate *vs*. Water then Milk	0.009639691	accepted
Control *vs*. Lactate then Milk	7.82 E−05	accepted
Control *vs*. Lactate	7.82 E−05	accepted
Control *vs*. Milk	0.999989867	rejected
Control *vs*. Water	0.855752344	rejected
Control *vs*. Water then Milk	7.82 E−05	accepted
Lactate then Milk *vs*. Lactate	0.016849546	accepted
Lactate then Milk *vs*. Milk	7.82 E−05	accepted
Lactate then Milk *vs*. Water	7.82 E−05	accepted

References

1. All about You, Smooth Chicken Liver Pate. Available online: http://www.allaboutyou.com/food/recipefinder/smooth-chicken-liver-pate-recipe-55329 (accessed on 17 March 2015).
2. Chicken Liver Parfait, Raymond Blanc's Kitchen Secrets. Available online: http://www.bbc.co.uk/food/recipes/chicken_liver_parfait_53623 (accessed on 17 March 2015).

© 2015 by the authors; licensee MDPI, Basel, Switzerland. This article is an open access article distributed under the terms and conditions of the Creative Commons Attribution license (http://creativecommons.org/licenses/by/4.0/).
